# Editorial: Perioperative hemodynamic monitoring and management

**DOI:** 10.3389/fmed.2022.1096310

**Published:** 2022-12-09

**Authors:** Guo-wei Tu, Xavier Monnet, Antoine Vieillard-Baron, Nikola Dobrilovic, Kanhua Yin

**Affiliations:** ^1^Department of Critical Care Medicine, Zhongshan Hospital, Fudan University, Shanghai, China; ^2^Paris-Saclay University Hospitals, Bicêtre Hospital, Inserm EA S_999, Paris-Saclay University, Paris, France; ^3^Hôpital Ambroise Paré, Assistance Publique Hôpitaux de Paris, Paris, France; ^4^Division of Cardiac Surgery, NorthShore University HealthSystem, Chicago, IL, United States; ^5^Department of Surgery, Washington University School of Medicine, St. Louis, MO, United States

**Keywords:** hemodynamic monitoring, fluid responsiveness, extracorporeal membrane oxygenation, sedation, Enhanced Recovery After Surgery

In recent years, perioperative hemodynamic monitoring techniques have made great strides as the accelerating introduction of non-invasive monitoring instruments such as critical care ultrasound (1, 2). As performing standardized hemodynamic monitoring is a significant routine for clinicians with respective specialties in intensive care unit, these strides enable them to produce better hemodynamic status observation, then provide timely while targeted interventions, and thereby improve patient prognosis. Furthermore, the concept of resuscitation has also undergone a radical transformation, changing from a cardiac output-centered strategy into a perfusion-driven strategy (3, 4).

On the Research Topic, prominent experts in the field of hemodynamic monitoring have been commissioned as guest editors. Also, clinicians and researchers worldwide are invited to present their latest findings and reflections, helping further the understanding of this pivotal theme. We hope to take this opportunity to bring the readers an introduction to the progress made in recent years in the aspect of perioperative hemodynamic monitoring and enhance current clinical practice.

We identified all nine papers on the Research Topic of “*Perioperative hemodynamic monitoring and management*” (https://www.frontiersin.org/research-topics/31049/perioperative-hemodynamic-monitoring-and-management), with 7 original research, 1 review, and 1 perspective. The topic focused on the most compelling spots of perioperative hemodynamic monitoring, including sedation, vasoactive medication usage, fluid responsiveness, goal-directed hemodynamic optimization protocol, and extracorporeal circulation support.

Fluid resuscitation has proven to be one of the most fundamental while crucial interventions for patients with circulatory failure (5). Two studies investigated the prediction of fluid responsiveness. Zhao et al. demonstrated that for patient who sustain spontaneous breathing, ΔCI >7.5% induced by unilateral PLR is able to predict fluid responsiveness in a comparative accuracy, which might bring amelioration for patients who cannot undergo bilateral PLR. Morakul et al. reported that a reduction in perfusion index (PI) during lung recruitment maneuver (LRM) and the baseline pulse pressure variation (PPV) are better predictors of fluid responsiveness for patients who underwent elective open abdominal surgery than the baseline cardiac output (CO), mean arterial pressure (MAP) and pleth variation index (PVI). These studies provided novel methods to optimize perioperative fluid administration.

Sedation plays an indispensable role in hypotension during the operation. Qiu et al. conducted a prospective controlled study on patients undergoing endoscopic submucosal dissection. The research compared hemodynamic stability of the patients who received propofol or remimazolam bolus induction and thereafter intravenous infusion. The outcome indicated that remimazolam clinically and statistically reduced the occurrence of peri-anesthesia hypotension. The superiority of remimazolam in maintaining hemodynamic stability and preventing hypotension may partially contribute to its preferable preservation of CO. Additionally, since whether patient height is associated with the block level for spinal anesthesia remains controversial, Huang et al. demonstrated that without prophylactic fluid preloading or vasopressors the dosing algorithm of bupivacaine based on height provided sufficient anesthesia with an infrequent occurrence of hypotension.

Accurate prediction of outcomes in critically ill patients is essential for clinical research and monitoring care quality (6). Xing et al. established a clinical information and image parameters based outcomes prediction model for the in-hospital patients with acute aortic dissection (AAD) from emergency department. The team identified that age, Marfan syndrome, type A aortic dissection, surgical repair, and maximum false lumen diameter are vital influencing factors on the in-hospital AAD outcomes. The end product named 3-level type A aortic dissection clinical prognosis score (3ADPS) proved to be able to provide rapid and effective prediction of in-hospital prognosis of type A aortic dissection. Low cardiac output syndrome (LCOS) after cardiac surgery may result in tissue malperfusion, as well as dysfunction of multiple organs including the liver, kidney, lung, brain and gastrointestinal tract. To twist the situation of lacking machine learning prediction model for the occurrence of LCOS after cardiac surgery, Hong et al. constructed several LCOS predictive models employed with respective machine learning algorithms, which may help to stratify the risk factors, detect the emergence early, and ultimately improve the LCOS management.

Post-operative delirium is commonly reported among aged patients with increasing morbidity and mortality. Whether the perioperative goal-directed hemodynamic optimization algorithm could make improvements in cerebral oxygenation and delirium prevention remains controversial. Fuest et al. reported that the application of an algorithm in high-risk noncardiac surgical patients failed to make any progress in hemodynamic interventions, hemodynamics amelioration, and cerebral oxygenation increment. Any effect brought by the trial on the occurrence of post-operative delirium remains undetected. With regard to venous-arterial extracorporeal membrane oxygenation (V-A ECMO), the critical circulatory support apparatus to rescue patients from refractory cardiogenic shock. During the V-A ECMO weaning phase, to simulate diverse loading conditions and assess the ventricles performance, the V-A ECMO centrifugal pump flow requires continuous adjustment (7). Luo et al. reported a novel method to evaluate cardiac function during V-A ECMO support (Flow challenge test, FCT). During the test, afterload will be converted into preload which can lead to a hemodynamic challenge, and by extension, the FCT result is reliable to predict the CO value after V-A ECMO weaning.

As the core of Enhanced Recovery After Surgery (ERAS) protocols, the goal-directed fluid therapy (GDT) concept has aroused growing attention around the world. Dmytriiev et al. presented the analytical data, giving a comprehensive description of the perioperative targeted infusion therapy benefits and principles to reduce the peril of complications on a central hemodynamic parameters based foundation. Furthermore, the encouraging outcomes of GDT practice inspired clinicians to continue the research on a central hemodynamics monitoring basis to optimize benefits of surgical patients.

In summary, this Research Topic collected a series of research and review articles related to “*Perioperative hemodynamic monitoring and management*”. All authors, reviewers, and editors who contributed are greatly appreciated. We hope that readers will enjoy this Research Topic, and the series will provide interested readers with new insights and inspiration for future research.

## Author contributions

G-wT drafted the manuscript. XM, AV-B, ND, and KY edited the manuscript, contributed to the Research Topic, and approved the publication of this Editorial. All authors contributed to the article and approved the submitted version.
